# Modified Star Excursion Balance Test Performance in Individuals With Chronic Ankle Instability: A Participant‐Level Analysis

**DOI:** 10.1002/jfa2.70115

**Published:** 2026-01-07

**Authors:** Lauren Forsyth, Jeffrey Simpson, Fereshteh Pourkazemi, Danielle M. Torp, Christopher Burcal, Rachel M. Koldenhoven, Luke Donovan, Abbis Jaffri, Saeed Al Adal, Andrew Mitchell, Craig R. Childs

**Affiliations:** ^1^ Faculty of Biomedical Engineering University of Strathclyde Glasgow UK; ^2^ Department of Movement Sciences and Health University of West Florida Pensacola Florida USA; ^3^ Sydney School of Health Sciences, Faculty of Medicine and Health The University of Sydney Sydney Australia; ^4^ College of Health Sciences Sports Medicine Research Institute University of Kentucky Lexington Kentucky USA; ^5^ School of Health and Kinesiology University of Nebraska at Omaha Omaha Nebraska USA; ^6^ Department of Health and Human Performance Texas State University San Marcos Texas USA; ^7^ Department of Applied Physiology, Health, and Clinical Sciences University of North Carolina at Charlotte Charlotte North Carolina USA; ^8^ Department of Physical Therapy Creighton University Omaha Nebraska USA; ^9^ Faculty of Applied Medical Sciences Najran University Najran Saudi Arabia; ^10^ Institute for Sport and Physical Activity Research University of Bedfordshire Bedford UK

**Keywords:** balance, chronic ankle instability, rehabilitation, star excursion balance test

## Abstract

**Introduction:**

For 1 in 5 individuals, an initial ankle sprain results in chronic ankle instability (CAI). Research is inconclusive as to whether individuals with CAI display decreased stability performance. This study conducted a participant‐level analysis to determine normative values for modified Star Excursion Balance Test (mSEBT) performance in a CAI population.

**Design:**

Participant‐level analysis.

**Methods:**

Ten datasets of mSEBT data were combined and participants categorised into one of three groups: individuals with CAI, Copers and Healthy participants. Maximal reach distances were analysed in the anterior (ANT), posterolateral (PL), posteromedial (PM) and composite (COMP) directions. The CAI and Healthy groups were sub‐categorised into 4 groups depending on the stance position and whether the average or best scores were reported.

**Results:**

The final pooled data consisted of 429 participants (202 CAI; 181 Healthy; 46 Coper). The 4 CAI sub‐groups recorded a mean reach of 66.53%–76.42% (ANT), 54.67%–87.16% (PM), 44.55%–83.01% (PL) and 55.25%–82.19% (COMP). Smaller reach distances were reported in Group 1's ANT, PL and COMP reaches and Group 2's ANT reach (*p* < 0.05) compared to the Healthy group. Copers exceeded CAI and Healthy reaches for all reaches. Reach distances in the ANT direction were generally the smallest for the CAI group and ANT and PL directions for the Healthy and Coper groups.

**Conclusion:**

Reduced mSEBT reach was performed in specific directions for specific sub‐groups only for the CAI population compared to both Healthy and Coper. The normative data can inform clinical practice and aid decision‐making regarding dynamic balance for assessment and rehabilitation purposes.

## 1 Introduction

Lateral ankle sprains are the most common lower limb injury, with 50% occurring during sport and physical activity and 50% occurring during other activities of daily life [1, 2, 3]. The risk of re‐sprain increases nine‐fold following one ankle injury [4], and for 1 in 5 individuals, an initial ankle sprain results in chronic ankle instability (CAI) [5, 6]. CAI is a multi‐faceted clinical condition associated with recurrent ankle sprains, pain, mechanical laxity and/or perceived instability [7]. The long‐term physiological and psychological effects of chronic conditions cause a substantial burden on an individual's ability to work and participate in physical activity, therefore negatively affecting quality of life [7]. Early diagnosis and intervention and continued assessment of CAI are critical for improving patients' musculoskeletal health and optimising rehabilitation strategies.

Balance is an essential requirement for all motor tasks, with balance involved when performing most movements in daily activities [8]. To assess balance for identifying injury risk and monitoring rehabilitation outcomes, it is critically important to have meaningful assessment tools. Various measurements and assessment techniques have evaluated balance following ankle sprains and for individuals with CAI [9, 10, 11]. However, the research has lacked consistency on measurement properties with static balance including the Balance Error Scoring System, Time in Balance Test, foot lift test, centre of pressure, instrumented wobble board (i.e., Biodex Balance System) and single leg heel raise [12, 13]. Dynamic balance has been reported using the Star Excursion Balance (SEBT), side hop, Dynamic Leap and Balance, step down and Figure of 8 hopping tests [4, 12, 14].

For individuals with CAI, the SEBT remains the recommended and most utilised measure of dynamic stability [15, 16, 17, 18]. During the test, participants are required to reach maximally in 8 directions while maintaining a unilateral stance on the opposite leg. One reason for the popularity of the SEBT is its easy implementation as an inexpensive clinical test while producing meaningful results [ICC 0.67–0.97] [19, 20]. For individuals with CAI, the SEBT has frequently been reported in previous research and meta‐analyses to highlight postural‐control deficits [21, 22, 23, 24]. Both Schurz et al..’s [10] and Hegedus et al..’s [25] systematic reviews reported that the SEBT was an effective tool to measure dynamic balance. Specifically, Hegedus and colleagues [25] reported the SEBT to be the only dynamic assessment analysed to consistently identify increased lower extremity injury risk among sporting populations, including the ankle. However, we note that there is also conflicting evidence showing no deficits to balance for individuals with CAI, with studies reporting that SEBT performance did not significantly differ between CAI and Healthy participants [8, 22, 26, 27, 28].

More recently, the modified SEBT (mSEBT) has been presented as a simplified yet highly reproducible version of the SEBT due to task redundancy of the SEBT [19, 29, 30]. The mSEBT has positively identified those with CAI in the previous literature [17, 30, 31], with a combined average calculated giving a composite score [19]. The mSEBT utilises only three of the eight reach directions—anterior, posteromedial and posterolateral [17, 19, 29, 30, 31]. Another common dynamic balance assessment is the Y‐Balance Test, and although these tests appear similar, there are technical differences that limit the ability to combine results [32]. For the mSEBT after the 5 directions were removed from the original SEBT, the angles between the 3 remaining directions are not consistent or configured to a 360° circle (resulting in 135° between anterior and posterior directions and 90° between the two posterior directions). The Y‐Balance Test is built from the 360° divided into 3 parts (therefore, 120° between each direction). Furthermore, the Y‐Balance Test uses a commercial setup where a block is pushed along a pipe, whereas for the mSEBT, the individual has their reaching limb open, where they are tasked to simply reach as far as possible and then lightly touch the ground in the respective direction. Due to these technical differences, this paper reports on the normative data for the mSEBT only.

Currently, there is no normative data reported for the CAI population for the SEBT or mSEBT. It is clinically important to quantify dynamic balance; thus, normative values will provide valuable information to clinicians, coaches, scientists and researchers for sports injury prevention and management. Recent focus has been placed on participant‐level analysis that enables larger sample sizes to capture the heterogeneity of CAI [33, 34]. Heterogeneity within small‐scale studies could explain mixed findings of differences between individuals with and without CAI, thus highlighting the need for normative data references.

Therefore, the primary aim of this study was to establish normative values for mSEBT performance among individuals with CAI, Copers and Healthy controls, using a participant‐level pooled analysis. In this study, performance on the mSEBT is defined as the maximum reach distance. As stated above, normative values are currently unknown; however, we hypothesise that mSEBT scores for those with CAI will be smaller than both Coper and Healthy controls.

## 2 Methods

### 2.1 Study Design

This study is a participant‐level analysis of data from 10 separate datasets of mSEBT data in a CAI, Coper and/or Healthy populations [35, 36, 37, 38, 39, 40, 41, 42, 43, 44]. All studies included were approved by the respective university ethics committees, and for each dataset, informed consent was obtained and the rights of participants were protected. All data were anonymised prior to secondary use and pooling of datasets. Data spanned across Australia, United Kingdom and the United States of America as the group of international researchers compiled data from respective projects. The process of data collection is provided in detail in the published studies and displayed in Table 1.

**TABLE 1:** Summary of the included study protocols.

	Number of CAI	Number of healthy	Number of coper	Foot placement	Best or average	Number of practice trials	Population (age, athletes)
Al Adal, S [34]	51	30	0	Heel for PM and PL and toe for A	Best	3 practice 3 test	Convenience sample (via University of Sydney bulletin boards)
Burcal et al. [35]	11	20	0	Midfoot in the centre and always pointing forward	Average	4 practice 3 test	Physically active adults
Burcal et al. [36]	21	0	0	Heel for PM and PL and toe for A	Average	4 practice 3 test	Physically active adults
Donovan et al. [37]	15	15	0	Heel for PM and PL and toe for A	Average	6 practice 3 tests	Healthy, physically active young adults
Donovan et al. [38]	26	0	0	Heel for PM and PL and toe for A	Average	6 practice 3 tests	Young adults with CAI from a university and surrounding community
Forsyth et al. [39]	17	10	0	Heel for all directions	Best	4 practice 1 test	General population from local universities and surrounding communities
Jaffri et al. [40]	18	18	0	Heel for PM and PL and toe for A	Average	3 trials	College‐aged recreationally active individuals
Koldenhoven et al. [41]	30	0	0	Heel for PM and PL and toe for A	Average	3 trials	n/a
Pourkazemi et al. [42]	28	67	46	Heel for PM and PL and toe for A	Best	3 practice 3 test	Convenience sample (via University of Sydney bulletin boards and web‐based bulletins)
Simpson et al. [43]	0	21	0	Midfoot in the centre and always pointing forward	Average	3 trials	Healthy collegiate aged female participants

*Note:* Not all publications reported mSEBT results in the final report; however, all authors have confirmed data were collected during the associated projects.

Abbreviation: n/a: not applicable (i.e., study did not mention).

### 2.2 Participants

Participants were categorised into one of three groups: those with CAI, Copers and Healthy participants.

Participants with CAI were screened using the International Ankle Consortium guidelines [45] and were required to have experienced the following: (1) history of at least one significant ankle sprain, (2) more than three months since most recent injury prior to study participation, (3) history of previous episodes of ‘giving way’, recurrent sprain or ‘feelings of instability’ and (4) perceived ankle instability determined by a score of ≤ 25/30 on the Cumberland Ankle Instability Tool (CAIT) [46], ≥ 5 on the Ankle Instability Instrument (AII) [47], or ≥ 11 on the Identification of Functional Ankle instability (IdFAI) [48].

Participants were considered as Copers if they had sustained at least one ankle injury that occurred at least 12 months ago and do not exhibit CAI symptoms defined in this study as a CAIT score of > 25 [49].

Participants were included in the Healthy group if they had not sustained any previous ankle injuries and scored > 24 on the CAIT, < 5 on the AII or < 11 on the IdFAI.

Data for participants were removed from this analysis if the perception of stability score from the CAIT, AII or IdFAI was out with the bounds above and/or the participant had reported either no ankle sprain in the CAI group or had reported at least 1 ankle sprain in the Healthy group. The original exclusion criteria for each included study can be found here [35, 36, 37, 38, 39, 40, 41, 42, 43, 44, 50, 51]. Broadly, studies excluded individuals with other lower limb injuries, fractures, neurological impairments or any other health‐related diagnosis impacting participation.

### 2.3 Instrumentation and Protocol

The CAIT, AII or IdFAI were used as screening tools for the data and were therefore completed prior to undertaking the mSEBT. Demographic information (sex/age/body mass) was collected before mSEBT completion. Participants wore comfortable clothing and were barefoot for testing.

**FIGURE 1:**
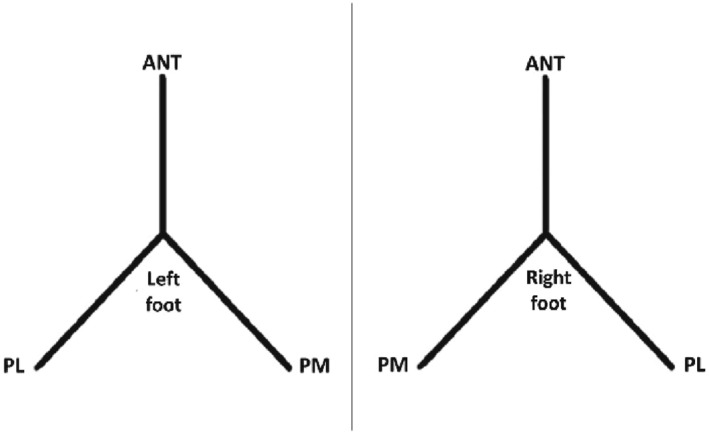
mSEBT reach directions to test the left foot (left) and right foot (right).

The mSEBT was completed with participants reaching to maximum distance in pre‐determined directions while maintaining unilateral stance and without shifting the weight from the supporting limb (Figure 1). Tape on the floor was there to guide the direction of each reach movement. Hands remained on the hips throughout the test. Loss of balance or failure to comply with the verbal instructions meant the trial was discarded and repeated. Across studies, participants were given 3–6 practice trials, to diminish learning effects, and three test trials [52]. The best or average performance across the three trials was analysed (Table 2) [35, 36, 37, 38, 39, 40, 41, 42, 43, 44, 50, 51].

**TABLE 2:** Sub‐categories for Groups 1–4 dependent on the stance position and whether average/best reach distance reported.

		Reach direction				
		Anterior	Posteromedial	Posterolateral		
Stance position	Group 1	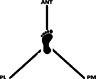	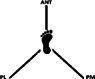	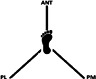	Average	Best or average reach distance reported
	Group 2	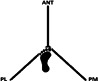	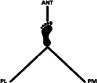	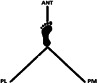	Average	
	Group 3	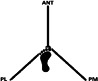	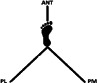	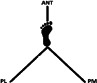	Best	
	Group 4	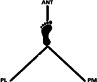	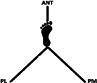	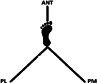	Best	

### 2.4 Data Analysis

Reach distances were analysed in the anterior (ANT), posterolateral (PL), posteromedial (PM) and composite (COMP—average of the three directions) directions. The maximal reach distance was defined as the furthest point reached by the big toe along the tape on the floor [19]. All data were normalised, and the reach distance was reported as a percentage of participant's leg length, which was defined as the distance between the ASIS and medial malleolus of the stance leg. Data were sub‐categorised based on the stance position [53] and whether the best or average reach distance was reported. This resulted in 4 sub‐categories for each CAI, Healthy and potential Coper group (Table 2). For each group the mean, median, standard deviation and 95% confidence intervals of the dependent variables were calculated, as well as the interquartile ranges of the reach distances.

### 2.5 Statistical Analysis

Statistical analyses were conducted using SPSS v26 (IBM, USA), and consistent with the Checklist for statistical Assessment of Medical Papers [54]. To account for variability across the pooled datasets, we conducted a linear mixed‐effects model (LMM) with the dataset entered as a random effect. Group (CAI, Coper, Healthy) was included as a fixed factor, and SEBT reach distances (anterior, posterolateral, posteromedial and average) were treated as continuous dependent variables. This approach estimated between‐study variance components, providing an assessment of inter‐study heterogeneity.

Independent‐samples t‐tests assessed demographic differences, whereas nonparametric tests evaluated patient‐reported outcomes. Group differences in the dependent variables were examined using a multivariate analysis of variance (MANOVA) to account for potential intercorrelations among outcome measures. When group (CAI, Healthy, Coper) and the sub‐group (1–4) were both controlled for, there were only differences for age and mass between the CAI and Coper Groups for 1 specific sub‐group for age and mass, respectively. For this reason, age and mass were not controlled for in the final reporting of results. Significance was set at *p* < 0.05 and partial eta squared (η^2^_p_) was used to estimate effect sizes (small: < 0.01; moderate: ∼0.06; large: > 0.14) [55, 56].

## 3 Results

### 3.1 Heterogeneity From Pooled Datasets

Inter‐study variance was reported modest but nonsignificant across all reach directions (ANT, PM and PL) and the resulting average of the three. The PL direction reported the greatest random intercept variance (*σ* ^2^ = 193.91, *p =* 0.068) compared to PM (*σ* ^2^ = 122.85, *p =* 0.071) Average (*σ* ^2^ = 86.67, *p =* 0.071) and ANT (*σ* ^2^ = 50.72, *p =* 0.076) in their respective order from largest to smallest variance. Therefore, we considered the datasets acceptable to pool for the participant‐level analysis.

### 3.2 Participant Demographics

The initial pooled data included 299 CAI participants, 201 Healthy participants and 46 Copers.

**FIGURE 2:**
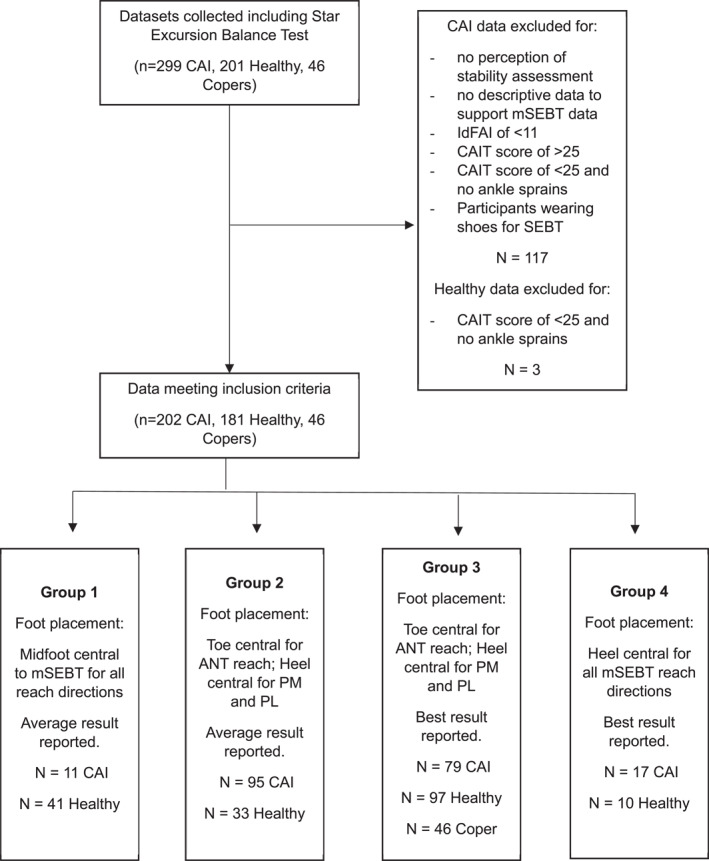
Data inclusion flow diagram.

From the CAI data, 80 files were removed for having either no perception of stability assessment or descriptive data to support mSEBT data, an IdFAI of < 11, a CAIT score of > 25 or a CAIT score of < 25 and no ankle sprains. Following this, a total of 202 participants remained for the analysis (Figure 2).

From the Healthy Group, 3 participants were removed for experiencing no ankle sprains but recording a CAIT score of < 25, leaving 198 participants.

The descriptive statistics are detailed in Table 3 for all participants. From the final pooled data of 429 participants (202 CAI; 181 Healthy; 46 Coper), 43.84% completed the CAIT, 9.59% completed the AII and 46.58% completed the IdFAI. Perceived stability of participants differed between the CAI and Healthy and CAI and Coper groups across all measures. No difference was reported between Coper and Healthy groups.

**TABLE 3:** Descriptive statistics for all CAI, Coper and Healthy participants.

	CAI	Coper	Healthy
Number of participants	202	46	181
Age (years)^b^ ^,^ ^c^	23.95 (6.32)	32 (8.53)	24.88 (7.56)
Mass (kg)^a^	71.56 (15.74)	67.37 (12.79)	66.81 (13.36)
Sex (F/M)	128/91	20/26	119/79
CAIT score^a^ ^,^ ^b^	18.42 (5.30)	28.85 (1.23)	29.12 (1.35)
AII score	6.62 (1.32)	n/a	n/a
IdFAI score^a^	20.29 (5.11)	n/a	1.26 (2.01)

*Note:* Results are presented as mean (SD) for continuous data and number for dichotomous data.

Abbreviations: AII: Ankle Instability Instrument; CAIT: Chronic Ankle Instability Tool; F: Female; IdFAI; Identification of Functional Ankle Instability; M: Male.

^a^ *p* < 0.05 between CAI and Healthy.

^b^ *p* < 0.05 between CAI and Copers.

^c^ *p* < 0.05 between Healthy and Copers.

The pooled data were sub‐categorised dependent on (1) stance position and (2) whether the best or average score of trials was reported (Figure 2 and Table 2). The best score of the trials (i.e., longest reach recorded) was reported for 168 participants and average across all trials reported for 261 participants. As stated in the methods, there were 3 stance positions used across the datasets. The most used foot placement was the toe central for the anterior reach and heel central for the PM and PL reaches (*n* = 174 CAI, *n* = 130 Healthy and *n* = 46 Copers). Twenty‐seven participants (*n* = 17 CAI, *n* = 10 Healthy) had their heel central for reaches to all directions. It should be noted that this heel stance only differed to the most used stance in the anterior direction as the heel position for the PM and PL directions was the same. The midfoot central stance was used for 11 CAI participants and 47 Healthy participants.

Once categorised into the 4 groups, the mean age (SE) for Groups 1 and 2 was significantly younger than Groups 3 and 4 for the population with CAI (Group 1: 20.91 ± 1.72 years; Group 2: 21.34 ± 0.59 years; Group 3: 27.09 ± 0.64 years; Group 4: 28.77 ± 1.39 years). The same differences were seen for the Healthy population where Groups 1 and 2 were significantly younger than Groups 3 and 4 (*p <* 0.05) (Group 1: 21.44 ± 1.10 years; Group 2: 21.61 ± 1.23 years; Group 3: 28.04 ± 0.72 years; Group 4: 28.40 ± 2.23 years).

### 3.3 Multivariate Analysis of Variance for Normative Data

A multivariate analysis of variance revealed significant main effects of the population (CAI, Coper or Healthy) (Pillai's Trace = 0.05, *F* (8, 836) = 2.68, *p* = 0.007, partial *η* ^2^ = 0.025) and the sub‐groups (Groups 1–4) (Pillai's Trace = 0.69, *F* (12, 1257) = 31.23, *p* < 0.001, partial *η* ^2^ = 0.230). Across the CAI, Coper and Healthy groups, this interaction was small; however, the sub‐group had a large interaction therefore suggesting a greater difference in the outcome based on the foot placement and whether the best or average result was reported.

However, although controlling for both variables incurred a significant population × sub‐group interaction (Pillai's Trace = 0.11, *F* (12, 1257) = 4.08, *p* < 0.001, partial *η* ^2^ = 0.037), the effect size was again small. Further analysis of this is reported in the following sub‐sections.

### 3.4 Comparison of Normative Values for mSEBT Across CAI, Coper and Healthy Groups

**FIGURE 3:**
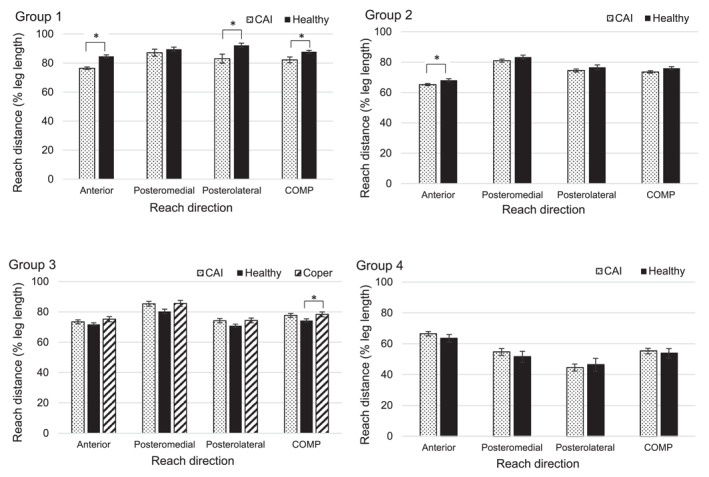
Mean (bars) and standard error (error bars) for each reach direction and COMP (average of the three directions) for each group (see Figure 2 for groups). Means adjusted for age as a covariate across CAI, Healthy and Coper. *p < 0.05 between participant groups.

Normative results for the mSEBT are presented in Figure 3 and Tables 4 to 5, 6.

**TABLE 4:** Descriptive statistics for CAI participants across all reach directions and composite scores.

CAI			Group 1 (*n* = 11)	Group 2 (*n* = 95)	Group 3 (*n* = 79)	Group 4 (*n* = 17)
ANT^†^ ^,^ ^*^ ^,^ ^a^ ^,^ ^c^	Mean (SE)		76.42 (0.9)	65.19 (0.7)	73.45 (1.3)	66.53 (1.4)
	Median		76.09	64.23	73.14	66.87
	IQ1		75.27	60.82	66.48	61.11
	IQ3		78.60	70.54	78.95	70.60
	95% CI	LB	74.32	63.82	70.88	63.51
		UB	78.51	66.57	76.03	69.55
PM^a^ ^,^ ^b^ ^,^ ^c^	Mean (SE)		87.16 (2.4)	80.94 (1.0)	85.34 (1.6)	54.67 (2.2)
	Median		89.01	81.30	84.33	55.37
	IQ1		80.46	74.07	77.39	46.70
	IQ3		93.60	87.84	92.82	60.29
	95% CI	LB	81.90	79.05	82.10	50.00
		UB	92.42	82.83	88.57	59.34
PL^a^ ^,^ ^b^ ^,^ ^c^	Mean (SE)		83.01 (3.1)	74.49 (1.0)	74.14 (1.4)	44.55 (2.3)
	Median		83.86	74.75	73.50	45.26
	IQ1		77.78	66.86	68.00	37.13
	IQ3		90.53	82.34	80.50	52.21
	95% CI	LB	76.06	72.44	71.35	39.64
		UB	89.96	76.54	76.94	49.46
COMP^†^ ^,^ ^*^ ^,^ ^a^ ^,^ ^b^ ^,^ ^c^	Mean (SE)		82.19 (2.0)	73.54 (0.8)	77.65 (1.3)	55.25 (1.8)
	Median		84.99	73.31	77.69	56.01
	IQ1		78.53	67.54	71.94	48.34
	IQ3		87.49	79.42	83.39	61.70
	95% CI	LB	77.79	71.98	75.05	51.36
		UB	86.60	75.10	80.24	59.14

*Note:* All results are a % of the participant leg length. Means adjusted for age across the sub‐groups.

Abbreviations: ANT: Anterior; CI: Confidence Interval; COMP: Composite; LB: Lower Bound; Max: Maximum; Min: Minimum; PL: Posterolateral; PM: Posteromedial; UB: Upper Bound.

^†^ *p* < 0.05 between Group 1 and Group 2.

^*^ *p* < 0.05 between Group 2 and Group 3.

^a^ *p* < 0.05 between Group 1 and Group 4.

^b^ *p* < 0.05 between Group 2 and Group 4.

^c^ *p* < 0.05 between Group 3 and Group 4.

^d^ *p* < 0.05 between Group 1 and Group 3.

**TABLE 5:** Descriptive statistics for Healthy participants across all reach directions and composite scores.

Healthy			Group 1 (*n* = 41)	Group 2 (*n* = 33)	Group 3 (*n* = 97)	Group 4 (*n* = 10)
ANT^†^ ^,^ ^a^ ^,^ ^c^ ^,^ ^d^	Mean (SE)		84.60 (1.0)	68.09 (1.0)	71.79 (1.0)	63.42 (2.5)
	Median		84.54	67.83	70.17	65.40
	IQ1		79.56	63.13	64.65	55.38
	IQ3		89.43	71.30	78.81	70.85
	95% CI	LB	82.59	66.00	69.78	57.71
		UB	86.62	70.18	73.80	69.15
PM^a^ ^,^ ^b^ ^,^ ^c^ ^,^ ^d^	Mean (SE)		89.46 (1.5)	83.33 (1.3)	80.28 (1.5)	51.49 (3.6)
	Median		90.85	83.80	77.34	55.99
	IQ1		84.62	78.29	70.55	41.88
	IQ3		96.29	88.12	90.24	59.82
	95% CI	LB	86.35	80.74	77.22	43.38
		UB	92.58	85.92	83.33	59.60
PL^†^ ^,^ ^a^ ^,^ ^b^ ^,^ ^c^ ^,^ ^d^	Mean (SE)		92.19 (1.5)	76.57 (1.6)	70.85 (1.1)	46.31 (4.2)
	Median		92.22	74.71	70.75	47.11
	IQ1		85.11	71.38	64.27	38.62
	IQ3		99.05	84.21	77.61	58.41
	95% CI	LB	89.24	73.21	68.66	36.86
		UB	95.13	79.93	73.03	55.75
COMP^†^ ^,^ ^a^ ^,^ ^b^ ^,^ ^c^ ^,^ ^d^	Mean (SE)		87.75 (1.0)	76.00 (1.0)	74.30 (1.0)	53.74 (3.1)
	Median		88.16	76.54	73.96	56.11
	IQ1		82.70	70.87	67.00	45.84
	IQ3		92.96	79.81	80.49	61.98
	95% CI	LB	85.80	73.86	72.24	46.65
		UB	89.70	78.13	76.37	60.84

*Note:* All results are a % of the participant leg length. Means adjusted for age.

Abbreviations: ANT: Anterior; CI: Confidence Interval; COMP: Composite; LB: Lower Bound; Max: Maximum; Min: Minimum; PL: Posterolateral; PM: Posteromedial; UB: Upper Bound.

^†^ *p* < 0.05 between Group 1 and Group 2.

*p < 0.05 between Group 2 and Group 3.

^a^ *p*< 0.05 between Group 1 and Group 4.

^b^ *p* < 0.05 between Group 2 and Group 4.

^c^ *p* < 0.05 between Group 3 and Group 4.

^d^ *p* < 0.05 between Group 1 and Group 3.

**TABLE 6:** Descriptive statistics for Healthy participants across all reach directions and composite scores.

Copers			Group 3 (*n* = 46)
ANT	Mean (SE)		75.17 (1.7)
	Median		73.93
	IQ1		65.85
	IQ3		84.89
	95% CI	LB	71.74
		UB	78.60
PM	Mean (SE)		85.57 (2.0)
	Median		84.11
	IQ1		76.08
	IQ3		96.54
	95% CI	LB	81.61
		UB	89.53
PL	Mean (SE)		74.36 (1.6)
	Median		74.25
	IQ1		67.65
	IQ3		83.25
	95% CI	LB	71.03
		UB	77.69
COMP	Mean (SE)		78.37 (1.6)
	Median		78.68
	IQ1		71.34
	IQ3		87.65
	95% CI	LB	75.17
		UB	81.56

*Note:* All results are a % of the participant leg length. Means adjusted for age.

Abbreviations: ANT: Anterior; CI: Confidence Interval; COMP: Composite; LB: Lower Bound; Max: Maximum; Min: Minimum; PL: Posterolateral; PM: Posteromedial; UB: Upper Bound.

Generally, the participants with CAI did not reach as far as the participants in the Healthy or Coper groups (Figure 3). For the ANT direction, mean reach distance differences of −8.53% (Group 1), −2.96% (Group 2), −1.44% (Group 3) and +2.98% (Group 4) were recorded, where a negative result showed that participants with CAI reached less than Healthy participants. For the PM direction, data reported differences of −2.02% (Group 1), −2.46% (Group 2), +4.85% (Group 3) and +3.00% (Group 4). For the PL direction, differences were −8.84% (Group 1), −2.15% (Group 2), +3.12% (Group 3) and −1.89% (Group 4). This led to COMP differences of −5.49% (Group 1), −2.53% (Group 2), +3.13% (Group 3) and +1.36% (Group 4) between participants with CAI and those without. This difference reached significance between the CAI and Healthy groups for Group 1's ANT, PL and COMP reaches and Group 2's ANT reach (*p* < 0.05). As seen with the positive differences above, Groups 3 and 4 reported a larger maximal reach distance in the group with CAI compared to the Healthy participant's reach distances; however, this difference did not reach significance. Copers (from Group 3) exceeded CAI and Healthy reaches across all directions (*p* > 0.05) and COMP (*p* < 0.05 between Copers and Healthy).

### 3.5 Sub‐Group Effects on mSEBT

Tables 4 and 5 report the reach distances for each group for participants with CAI and the Healthy group, respectively. Group 1 recorded the furthest reaches across all directions for both CAI and Healthy participants out of the 4 groups, and Group 4 recorded the smallest reaches across all directions out of the 4 groups. For both participants with CAI and Healthy participants, the different foot positions and method of reporting resulted in significant differences. This occurred in the ANT direction and COMP where the groups all significantly differed from one another. Group 4 was the only group to significantly differ to all groups across all reach directions and COMP.

For Groups 1–3 of the CAI participants, the smallest reach occurred in the ANT direction and the largest in the PM direction. For Group 4, the smallest reach occurred in the PL direction and the largest in the ANT.

For the Healthy participants, the smallest reach direction was the ANT for Groups 1 and 2, and the PL direction for Groups 3 and 4. The greatest reaches were seen on average in the PL direction for Group 1, PM direction for Groups 2 and 3 and ANT for Group 4. Groups 1, 2 and 4 were consistent across the CAI and Healthy participants of which directions recorded the smallest and greatest reaches.

For Copers (from Group 3 only), the smallest reach distance was recorded in the PL direction and largest in the PM (Table 6).

## 4 Discussion

The mSEBT remains the recommended test for dynamic stability in individuals with CAI according to clinical guidelines published in 2016 [6, 57, 58]. In addition to being a screening tool, the mSEBT is used to measure improvement following intervention [59, 60]. Normative values have yet to be reported for a larger CAI population dataset. This is clinically important for applied practice, providing a mean performance standard for the clinical population. This can assist in the clinical decision‐making process.

Generally, the participants with CAI did not reach as far as the participants in the Healthy or Coper groups, partially supporting previous research and our original hypothesis [8, 9, 21, 23, 61, 62]. Specifically, CAI reach performance was significantly less than the Healthy group for Group 1's ANT, PL and COMP reaches and Group 2's ANT reach. As a result CAI reach performance was dependent not only on the sub‐group but also the reach direction [8, 9, 21, 23, 61, 62].Song and colleagues (2022) recently reported significantly reduced reach distance in 17/23 studies of their systematic assessing mSEBT performance in the CAI population. Of these 17 studies, the difference also resulted from specific directions rather than across all directions, supporting the results of this study. The remaining six studies of the review reported no differences between the CAI and Healthy groups [62], which has also been reported in other previous literature [27]. These publications supported Groups 3 and 4 in this study, which reported no significant differences between the CAI and Healthy participants.

From the results, the ANT was generally the worst performing direction for those with CAI. Gottleib et al.. [61] and Jaber et al.. [8] also reported the smallest reach distance to be in the ANT direction and to significantly differ from the Healthy control group. However, this contrasts with conclusions from the systematic review from Rosen et al.. [23], where the ANT and PL direction results were small and considered unimportant, concluding that those with shorter anteromedial, medial and posteromedial reach distances are more likely to have CAI. This is interesting since the mSEBT does not include the anteromedial or medial direction, and our study did not find any differences between the CAI and Healthy groups for the PM direction. Furthermore, the PM direction has been reported to best represent the mSEBT overall as the greatest hip and knee flexion are required for maximal reaching compared to the other reach directions [21]. However, our results show that it was the best performing reach distance for participants with CAI in 3 of the 4 sub‐groups, and for Group 4 it was neither the best nor the worst.

In this study, the Coper group displayed greater maximal reach distances than both the CAI and Healthy groups, although this did not reach significance. It should be noted that the sample size for the Coper group was a lot smaller than the CAI and Healthy groups in Group 3; however, recent literature has identified similar findings to this study [63]. Kwon [63] reported significantly greater reach distances in the Coper group compared to both a CAI and Healthy group in the PL direction. Doherty et al.. [21] reported a significantly greater reach in the Copers to the CAI group in all reach directions, however no difference to the Healthy group. Currently, there is no clear rationale for this result; however, it has been suggested that Copers develop compensatory mechanisms to aid movement efficiency and protect from re‐injury, which healthy individuals who have never had an ankle injury have not had to look to or develop [63]. This may be a neuromuscular compensation, as previous literature has found Copers, compared to Healthy, had increased muscle activation during the mSEBT without any difference in the reach distance [27]. As the mSEBT relies on a complex single‐leg squat and reach movement pattern, there are many factors that were not measured in the current study that could explain our findings. Future research should examine the biomechanical and neuromuscular differences of those with when performing the mSEBT. This would provide valuable insights into compensation strategies employed by individuals with CAI which, as a result, reduces the reach distance as the musculoskeletal system is employing a protective mechanism.

The results of the study identified the importance for consistent testing standards by the significant differences in reach distances across the 4 sub‐groups in both the CAI and Healthy groups. We found that the different foot positions, and method of reporting (best/average), resulted in significant differences for the ANT direction and COMP result in both CAI and Healthy groups. This supports the work by Cug [53] who highlighted the significant differences in reach distances for the different stance positions tested in a CAI‐only population.

### 4.1 Clinical and Research Implications

Small and insignificant differences between groups highlight the inefficacy of the mSEBT as a diagnostic screening tool. Despite these findings from our normative data, we feel the mSEBT still has utility as a clinical assessment tool. The International Ankle Consortium has endorsed employing an impairment‐based approach to assessment and rehabilitation of patients with CAI [16]. The SEBT was identified as a critical component of the International Ankle Consortium rehabilitation‐oriented assessment for assessing and tracking improvement dynamic stability throughout the rehabilitation process. The results from this study highlight the importance of consistent protocols in practice, and the normative values generated from over 200 participants with CAI could be used to benchmark rehabilitation or set goals when working with individual patients. This is particularly relevant for those with bilateral CAI, where inter‐limb symmetry comparisons cannot be made, and removes the assumption that the non‐CAI side performance is desirable. Further research is still warranted in establishing clinical cut‐offs such as the minimal clinically important difference in patients with CAI to aid in clinical decision‐making and to improve reporting of responder analyses in clinical trials.

### 4.2 Strengths and Limitations

The main strength of this study was the large sample population that represented a diverse group of both the general and athletic population internationally. This is compared to most CAI research in smaller numbers of youth or college athletes. Normative values for age groups could not be completed given that the majority of participants fell into the 20–30‐year‐old age group.

Given the manual assessment of the mSEBT, it can be difficult to accurately measure each maximal reach distance. In addition, the time needed to reach each target, the accuracy of the pointing tasks and the range of motion of the lower limb joints are all important contributing factors to balance but not assessed in the mSEBT [28].

This report has been transparent to the differences between the 10 datasets pooled for this participant‐level analysis. The participants were sub‐categorised depending on the stance position and method of reporting (i.e., best or average). The remainder of the protocols was consistent due to the standardisation and commonality of the mSEBT.

## 5 Conclusion

This study reports normative data for a large dataset of individuals with CAI, Copers and Healthy. Generally, results found a reduced mSEBT performance for individuals with CAI only in specific reach directions compared to groups with Healthy and Coper participants. The ANT and PL reach directions exhibited the poorest results for the CAI group. The normative values provide critical information to inform clinical decision‐making in practice. This will be particularly important during the assessment of dynamic balance and rehabilitation outcomes; however, further insights are required to understand the movement strategies utilised to achieve these reach distances and the neuromuscular and biomechanical differences between the CAI, Coper and Healthy populations.

## Author Contributions

**Lauren Forsyth:** conceptualization, data curation, writing – original draft, project administration, writing – review and editing. **Jeffrey Simpson:** conceptualization, data curation, writing – review and editing. **Fereshteh Pourkazemi:** data curation, writing – review and editing. **Danielle M. Torp:** conceptualization, writing – review and editing. **Christopher Burcal:** conceptualization, data curation, writing – review and editing. **Rachel M. Koldenhoven:** conceptualization, data curation, writing – review and editing. **Luke Donovan:** conceptualization, data curation, writing – review and editing. **Abbis Jaffri:** data curation, writing – review and editing. **Saeed Al Adal:** data curation, writing – review and editing. **Andrew Mitchell:** writing – review and editing. **Craig R. Childs:** conceptualization, project administration, writing – review and editing.

## Funding

The authors have nothing to report.

## Ethics Statement

All studies included in the analysis were approved by the respective University ethics committees with participants providing consent prior to participation.

## Conflicts of Interest

The authors declare no conflicts of interest.

## Data Availability

The data that support the findings of this study are available from the corresponding author upon reasonable request.
